# Impact of extraction methods on soybean hull polysaccharides: structure and functional properties analysis

**DOI:** 10.1016/j.fochx.2025.103049

**Published:** 2025-10-18

**Authors:** Rui Zhang, Daozi Deng, Hong Song, He Liu, Shuai Yang

**Affiliations:** a College of Food Science and Technology, Bohai University, Jinzhou 121013, China; b Grain and Cereal Food Bio-efficient Transformation Engineering Research Center of Liaoning Province, Jinzhou 121013, China; c Liaoning Province Key Laboratory for Emission Reduction, Efficiency Improvement and Comprehensive Utilization of Agricultural Products, Jinzhou 121013, China; d Beipiao Haifeng Food Industry Co. Ltd., Beipiao 122100, China

**Keywords:** Polysaccharides, Microwave, Extraction methods, Emulsifying

## Abstract

Soybean hull polysaccharides (SHP) exhibit diverse functional properties, yet the influence of extraction methods on their structure–function relationships remains unclear. This study investigated microwave-assisted extraction (M), extractant-assisted (citric acid, MC; sodium citrate, MS), and extractant-enzyme (cellulase/pectinase)-assisted (MCC, MCP, MSC, MSP). The results show that the M have a relatively high extraction yield (3.39 %) and carbohydrate content (92.64 %) compared with others. MS have the highest glucuronic acid content (41.60 %). Structural analysis revealed that M, MC, MCC, and MCP produced RG-I-type pectins with high branching degrees (12.85–16.71), whereas MS, MSP, and MSC generated predominantly linear homogalacturonan (HG) domains (linearity: 1.51–1.81). Moreover, Na^+^ combined with higher HG domains reduced electrostatic repulsion and promoted polysaccharide alignment at the oil–water interface, leading to superior emulsifying activity (277.48 m^2^/g) and stability (6232.07 min) in MS. These findings clarify SHP structure–function relationships and provide guidance for tailoring polysaccharide properties through optimized extraction strategies.

## Highlights

• Microwave pretreatment enhances the yield and carbohydrate content of SHP.
• Extraction methods significantly influence SHP's viscosity, monosaccharide content, and structure.
• MS-SHP exhibits superior emulsifying activity and emulsion stability.
• MS-SHP has primarily HG-type structural domains, while MC-SHP is mainly RG-I type.

## Graphical abstract

Investigating the effects of different extraction methods on the physicochemical properties, structural characteristics, and functional properties of soybean hull polysaccharides.

**Figure:**
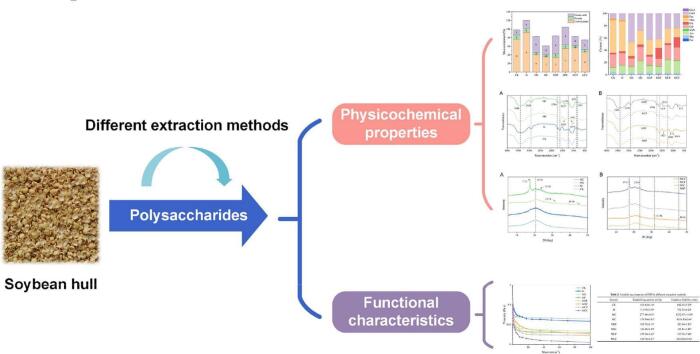

## 1 Introduction

Polysaccharides are high-molecular-weight carbohydrates composed of multiple monosaccharide units linked by glycosidic bonds. They are ubiquitous in nature, primarily present in plants, animals, and microorganisms (Netrusov et al., 2023), functioning as essential components for structural support and energy storage in biological systems. Natural polysaccharides exhibit significant structural diversity, with their distinct chemical architectures determining a wide range of biological activities and functional properties (Geng et al., 2024).

Soybean (*Glycine* max) is an annual herbaceous plant of the legume family, recognized for its rich nutritional value, making it a central subject of global agricultural research. Soybean hulls form the outer protective layer of soybean seeds and represent a major by-product of soybean processing, accounting for approximately 8 % of the total soybean weight (Han et al., 2021). Traditionally, soybean hulls have been used as animal feed or discarded as waste. Research indicates that soybean hulls are rich in pectin, cellulose, and hemicellulose, highlighting their significant utilization potential as raw materials for polysaccharides (Yang, Lei, et al., 2020). Soybean hull polysaccharides (SHP), a pectic polysaccharide extracted from soybean hulls, is rich in homogalacturonic acid (HG) and rhamnogalacturonan (RG-I). These polysaccharides are structurally complex anionic acidic compounds, consisting of various monosaccharides linked by O-glycosidic bonds. In addition to their structure similar to pectin, the side chains of these polysaccharides include α-1,5-L-arabinosyl, β-1,4-mannosyl, and β-1,4-D-galactosyl linkages (Han et al., 2021). SHP has been demonstrated to possess various functional properties, including antioxidant activity, emulsifying capability, and gelling properties. Additionally, they can serve as prebiotics for gut microbiota, aiding in the regulation of the gut microbiome. Despite their beneficial properties, the low extraction efficiency and poor water solubility of SHP limit their practical applications.

The extraction of polysaccharides serves as a fundamental step in investigating their biological activities. In recent years, people have conducted a lot of research and exploration on the extraction methods of the natural product polysaccharides (Yahaya et al., 2024). At present, the commonly used extraction methods are extractant extraction and enzyme extraction. Extractant extraction including water extraction, alkali extraction, and acid extraction (Zhao et al., 2023). Among them, water extraction is particularly favored (Wang et al., 2022). This method is simple and cost-effective, but prolonged intensive heating can degrade the polysaccharide structures within the cells, reducing both their bioactivity and yield. Enzymatic extraction is a gentle and efficient method for extracting polysaccharides. Compared to solvent extraction, it significantly reduces extraction time while enhancing the extraction yield. However, its high cost limits its widespread application. Microwave technology, as a form of thermal processing, has been extensively utilized in domestic and industrial food preparation (Yi et al., 2024). Microwave-assisted extraction possesses superior penetration capabilities, enabling a significant reduction in extraction time and energy consumption. Owing to these advantages, this technique has been widely adopted for the extraction of bioactive polysaccharides (Peng et al., 2024). The non-ionizing radiation of microwaves rapidly penetrates plant tissues, thereby enhancing the diffusion coefficient and mass transfer efficiency of polysaccharides during pretreatment via dipole interactions and the conductive migration of dissolved ions (Bamigbade et al., 2024). Furthermore, microwave-assisted extraction improves the yield of polysaccharides and modifies their structural and physicochemical properties compared to conventional extraction methods. The functional properties of polysaccharides are closely tied to their structural characteristics, including molecular weight, monosaccharide composition, glycosidic linkage patterns, α/β-configuration, branching patterns, and degree of substitution. These features are significantly influenced by extraction methods and conditions (Lv et al., 2024). Research highlights the crucial role of extraction techniques in determining polysaccharide yields and their functional properties (Jiao et al., 2023).

However, to date, research on SHP has primarily focused on improving extraction methods and conducting preliminary physicochemical property analyses. The mechanism by which microwaves in combination with extractants and enzymes influence the structure and functional properties of SHP remains unclear. Therefore, this research aimed: (a) to extract polysaccharides from soybean hull using 8 extraction methods: hot water extraction (CK), microwave-assisted extraction (M), extractant-assisted extraction (citric acid, MC; sodium citrate, MS), and extractant-enzyme (cellulase/pectinase)-assisted extraction (MCC, MCP, MSC, MSP); (b) to determine the emulsifying properties of SHP extracted by different methods, including relating these properties to structure; (c) to compare the emulsifying properties of microwave in combination with extractants and enzymes extracted polysaccharides with those of polysaccharides extracted by the conventional water method. The structural properties of the extracted polysaccharides were investigated by molecular weight, monosaccharide, FT-IR, XRD, AFM and SEM analysis. The results of this study lay the foundation for identifying polysaccharides with diverse structures and provide a theoretical basis for the preparation of soybean hull polysaccharides with distinct functional properties.

## 2 Materials and methods

### 2.1 Materials and chemicals

Soybean hulls were purchased from Yu Wang Group (Harbin Province, China). The soybean hulls were cleaned of impurities, dried in an oven at 65 °C for 1–2 h, crushed using a cyclone mill, and sieved through a 60-mesh screen. The soybean powder was decolorized with 1 % ethanol at a 1:10 ratio at room temperature, and the decolorized samples were dried at 65 °C before being sealed in self-sealing bags for storage. Sodium citrate and citric acid were purchased from Tianli Chemical Reagent Co., Ltd. Pectinase and cellulase were obtained from Pombo Biological Engineering Co., Ltd. All other chemicals and reagents were of analytical grade.

### 2.2 Extraction of soybean hull polysaccharides

#### 2.2.1 Microwave pretreatment for polysaccharide extraction

The polysaccharide extraction method was adapted from the literature (Han et al., 2021) with modifications. The soybean hulls were cleaned to remove impurities and then dried in an oven at 65 °C for 1–2 h. After drying, the hulls were ground using a cyclone mill and passed through a 60-mesh sieve. A 1 % ethanol solution was added at a 1:10 ratio, and the mixture was stirred at room temperature for 30 min for decolorization. The mixture was then filtered through double-layer gauze, and the filter residue was dried in an oven at 65 °C.

Based on the experimental approach of Yang et al. (Yang, Zhang, et al., 2020) and through pre-experimental exploration and improvement, the following parameter conditions for obtaining polysaccharides using different extraction methods were determined in this experiment. Soybean hull powder was mixed with distilled water at a solid-to-liquid ratio of 1:5 and then subjected to microwave pretreatment (480 W, 15 min). After cooling, water was added to adjust the solid-to-liquid ratio to 1:20 (g/mL). The mixture was extracted with hot water at 85 °C for 4 h. After cooling and filtration, the mixture was centrifuged at 4000 rpm for 10 min, and the supernatant was rotary evaporated to one-third of its original volume. The pH was adjusted to 4.0 using 1 M hydrochloric acid solution. The concentrated solution was slowly added to twice its volume of ethanol and left to precipitate at 4 °C for 12 h. Microwave-assisted extraction soybean hull polysaccharides (M) were obtained through lyophilization. Hot water extraction (CK) was conducted similarly to the above method, but without the microwave pretreatment process (solid-to-liquid ratio of 1:5, 480 W, 15 min).

#### 2.2.2 Microwave pretreatment combined with other methods of extraction

A 0.6 % (g/mL) solution of extractant (citric acid/citrate) at a 1:5 ratio was added to 50 g of soybean hull powder, followed by microwave pretreatment (480 W, 15 min). After cooling, water was added to adjust the solid-to-liquid ratio to 1:20 (g/mL). The mixture was then incubated in a water bath at 85 °C for 4 h, cooled, and filtered. The filtrate was centrifuged at 4000 rpm for 10 min, and the supernatant was evaporated to one-third of its original volume using a rotary evaporator. The pH was adjusted to 4.0 using 1 mol/L hydrochloric acid solution. Twice the volume of ethanol was slowly added to the concentrate and left to precipitate at 4 °C for 12 h. The precipitate was freeze-dried to obtain soybean hull polysaccharides (MC/MS). The polysaccharide extraction method was referred to Yang et al. (Yang, Liu, et al., 2023).

A 0.6 % (g/mL) solution of extractant (citric acid/citrate) at a 1:5 ratio was added to 50 g of soybean hull powder, followed by microwave pretreatment (480 W, 15 min). After cooling, water was added to adjust the solid-to-liquid ratio to 1:20 (g/mL). Then 2 % enzyme (pectinase/cellulase) was added, and enzymatic hydrolysis was carried out at 55 °C for 1.5 h, followed by incubation in a water bath at 85 °C for 2.5 h. After cooling, the solution was filtered and then centrifuged at 4000 rpm for 10 min. The supernatant was evaporated to one-third of its original volume using a rotary evaporator, and the pH was adjusted to 4.0 using a 1 M hydrochloric acid solution. Twice the volume of ethanol was slowly added to the concentrate, and the mixture was left to precipitate at 4 °C for 12 h. The precipitate was freeze-dried to obtain polysaccharide samples: citric acid – enzyme (pectinase/cellulase) assisted extraction polysaccharide (MCP/MCC), citrate - enzyme (pectinase/cellulase) assisted extraction polysaccharide (MSP/MSC).

### 2.3 Determination of carbohydrate, protein, and uronic acids in SHP

A total of 8 polysaccharide samples, each sample was repeated three times. The polysaccharide content was determined using the phenol‑sulfuric acid method The protein content in SHP was determined using Bicinchoninic Acid Assay (Yang, Zhang, et al., 2020). Uronic acid units were quantified using the colorimetric method after treatment with concentrated sulfuric acid and carbazole (Han et al., 2021).

### 2.4 Monosaccharide composition determination

Monosaccharide composition analysis was used for qualitative and quantitative analysis by an ion chromatograph equipped with a pulse amperometric detector, as previously reported (Bai et al., 2023). A total of 8 polysaccharide samples, 5 mg of each was hydrolyzed with 500 μL of sulfuric acid at 35 °C for 1 h. The hydrolyzed sample was then mixed with 2.5 mL of ultrapure water, thoroughly combined, and heated at 98 °C in a water bath for 1 h. After cooling to room temperature, the hydrolysate was adjusted to pH 7.0, diluted to a concentration of 20 mg/L, and filtered through a 0.45 μm membrane. The monosaccharide composition of SHP was analyzed using ion chromatography. Chromatographic conditions were as follows: CarboPac PA20 column (3.0 mm × 150 mm); injection volume: 10 μL; flow rate: 1.0 mL/min; mobile phases: A, 0.2 mol/L NaOH; B, 1 mol/L NaAC; C, DDH_2_O.

### 2.5 Fourier transform-infrared (FT-IR) analysis

A total of 8 polysaccharide samples, 1 mg of dried polysaccharide sample was mixed with KBr (100 mg) in an agate mortar, and the mixture was pressed into pellets for FT-IR analysis. The scanning range was set to 400–4000 cm^−1^ for the FT-IR spectral analysis. The methyl-esterification (DM) was calculated based on the peaks at 1740 cm^−1^ and 1630 cm^−1^ according to the following formula (Lv et al., 2024):

(1) $$\mathrm{DM}\left(\%\right)=\frac{A_{1740}}{A_{1630}-A_{1740}}\times 100$$

where A_1740_ is the peak area of methylated uronic acid group, and A_1630_ is the peak area of free uronic acid group.

### 2.6 X-ray diffraction (XRD) analysis

A total of 8 polysaccharide samples, each sample was repeated three times. The characterization of the SHP samples was performed using X-ray diffraction (XRD) with a current of 40 mA and a voltage of 40 kV, scanning over a diffraction angle (2θ) range from 5° to 50° at a rate of 5°/min (Wang et al., 2025).

### 2.7 Scanning electron microscopy (SEM) analysis

The 8 polysaccharide surfaces were coated with a thin layer of gold to enhance electrical conductivity and were subsequently examined using field emission scanning electron microscopy (SEM) at a magnification of 10,000× and an accelerating voltage of 2 kV (Song et al., 2022).

### 2.8 Emulsifying properties

To prepare the emulsion, the polysaccharide sample was dissolved in distilled water to obtain a 1 % (g/mL) solution. Soybean oil (10 % *v*/v) was then added, and the mixture was homogenized at 13,000 rpm for 2 min using a high-speed shear, resulting in a primary emulsion. This primary emulsion was subsequently passed through micro-jet equipment at 50 MPa for three cycles to produce the final emulsion, to which 0.02 % sodium azide was added as a preservative to inhibit microbial growth.

A total of 8 polysaccharide samples, each sample was repeated three times. Following the method described by Comas (Comas et al., 2006), the emulsion was prepared and then diluted 1000-fold with a 0.1 % sodium dodecyl sulfate (SDS) solution. A 0.1 % SDS aqueous solution was used as a blank control. The absorbance at 500 nm was measured using a UV spectrophotometer to calculate the emulsification activity index (EAI). After allowing the emulsion to rest for 30 min, the absorbance was measured again to determine the emulsification stability (ESI) using the following formula:

(2) $$\mathrm{ESI}\left(\min\right)=\frac{A_{0}}{A_{0}-A_{30}}\times 30$$

(3) $$\mathrm{EAI}\left(m^{2}/g\right)=2\times 2.303\frac{A_{0}\times N}{C\times \phi \times 10000}$$

Where A_0_ and A_30_, denote the absorbance values at 0 min and 30 min respectively. N is the number of dilutions. C is the polysaccharide concentration (g/mL). *φ* is the volume fraction of the oil phase in emulsion.

### 2.9 Viscosity analysis

The 8 polysaccharide samples were prepared as a 1 % (g/mL) solution and analyzed using a rheometer equipped with a 35 mm diameter circular steel plate. The parameters for measuring apparent viscosity included a temperature of 25 °C, a shear rate range of 0.1–100 s^−1^, and a gap setting of 0.5 mm(Liu, Wang, et al., 2024).

### 2.10 Statistical analysis

All experiments were performed in triplicate, and the data were expressed as the mean ± standard deviation. Graphical representation was done using Origin software, and statistical analysis was conducted using analysis of variance (ANOVA) in SPSS 19.0. A *p*-value of less than 0.05 was considered statistically significant.

## 3 Results and discussion

### 3.1 Effect of extraction method on SHP extraction yield

The extraction yield of SHP obtained by different extraction processes is shown in Table 1. Significant differences were observed among the different extraction groups (*p* < 0.05). MSP had the highest yield (13.32 %), followed by MSC (12.73 %), MS (11.90 %), MC (8.86 %), MCP (7.65 %), MCC (6.98 %), M (3.39 %) and CK (1.66 %). Compared with CK group, the extraction yield increased by 2.04 times after microwave pretreatment (M). The primary reason for the increased extraction yield is that microwave pretreatment, due to its penetrative ability, causes a rapid rise in the internal temperature of cells, leading to increased internal pressure, expansion of cell contents, and rupture of the cell wall. Moreover, the electric and magnetic effect can disrupt hydrogen bonds, affecting the solubility of polysaccharides and increasing the extraction rates.

**Table 1:** Extraction yield of SHP by different extraction methods.

Sample	Extraction yield (%)
CK	1.66 ± 0.16^f^
M	3.39 ± 0.46^e^
MS	11.90 ± 0.12^b^
MC	8.86 ± 0.65^c^
MSP	13.32 ± 0.34^a^
MSC	12.73 ± 0.21^a^
MCP	7.65 ± 0.28^d^
MCC	6.98 ± 0.11^d^

Data represent mean ± standard deviation (n = 3), and different letters indicate significant differences (p < 0.05) between different extractions.

The addition of extractants also significantly increased the extraction yield of polysaccharides. Specifically, the extraction yield increased by 7.17 times with sodium citrate (MS) and 5.34 times with citric acid (MC), indicating that sodium citrate is more effective than citric acid in enhancing the yield. The addition of pectinase and cellulase did not result in significant changes, although pectinase was slightly more effective than cellulase. The differences in extraction yield were attributed to the varying pH values of the extractants. SHP identity as an acidic polysaccharide results in the increased solubility in acidic environments. Additionally, the presence of metal ions in sodium citrate can effectively bind with cationic bonds in the polysaccharides, enhancing their water solubility and thus increasing the polysaccharide extraction yield (Zhang et al., 2020).

The extraction yields of both MSP and MSC increased with enzyme treatments, indicating that enzyme-assisted extraction can disrupt cell walls and break down complex intracellular compounds in the soybean hull, thereby enhancing polysaccharide release (Yang, Wang, et al., 2024). Tang et al. (Tang et al., 2023a) extracted polysaccharides from Dendrobium officinale using enzyme-assisted extraction and found that this method not only increased polysaccharide yield but was also effective in reducing oxidative stress levels in *Caenorhabditis elegans*. However, a decrease in the yield of MCP and MCC was observed, possibly due to the low pH of citric acid inhibiting enzyme activity (Edema et al., 2024).

### 3.2 Basic components of SHP

**Fig. 1:**
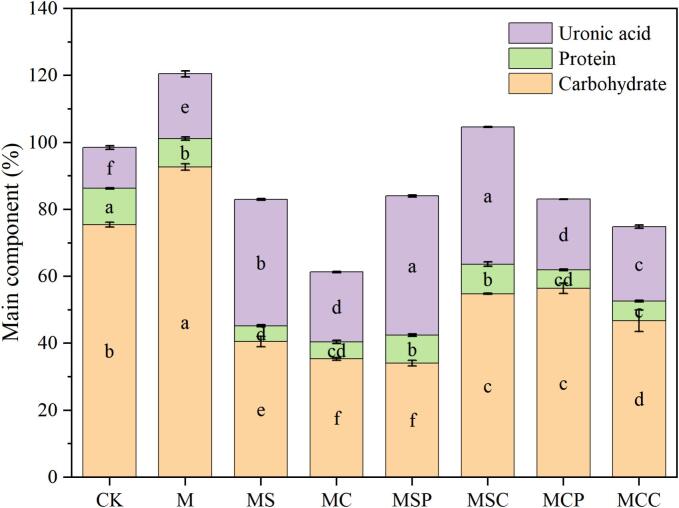
Basic fractions of SHP by different extraction methods. Data represent mean ± standard deviation (*n* = 3), and different letters indicate significant differences (*p* < 0.05) between different extractions.

Fig. 1 illustrates the main components of SHP extracted by different methods. Significant differences were observed in the carbohydrate content of SHP extracted by different methods (*p* < 0.05). After microwave pretreatment, the carbohydrate content of polysaccharides increased from 75.45 % ± 0.75 % to 92.64 % ± 0.94 %, indicating that microwave pretreatment significantly enhances carbohydrate content. Polysaccharides extracted with citric acid showed higher carbohydrate content than those extracted with sodium citrate, with MC and MCP having relatively high carbohydrate contents of 54.80 % ± 0.15 % and 56.47 % ± 1.56 %, respectively. MCC exhibited a lower content of 46.74 % ± 3.20 %, significantly different from MS, MSP, and MSC, which had contents of 40.55 % ± 1.52 %, 35.33 % ± 0.46 %, and 34.04 % ± 0.82 %, respectively. These results suggest that SHP extracted with acidic extractants has a significantly higher carbohydrate content than those extracted with near-neutral extractants.

Significant differences were also observed in the protein content of SHP extracted by different methods (p < 0.05). The CK group exhibited a significantly higher protein content (10.83 % ± 0.17 %) compared to the treatment groups, with the MS group showing a protein content of 4.69 % ± 0.31 %. The results suggest that extraction by microwave pretreatment can increase the polysaccharide content in crude polysaccharides and reduce the protein content. This effect may be due to the diverse structural types of constituents within the extract solution, each requiring a specific amount of microwave energy, which leads to variations in constituent content (Gu et al., 2024). Moreover, the inclusion of enzymes can increase the protein content in polysaccharides; however, the low pH of acidic extractants may cause protein denaturation. As a result, no significant increase in protein content was observed in MCP and MCC.

The MSP and MSC polysaccharides extracted from soybean hulls by different methods exhibited the highest uronic acid contents, at 41.60 % ± 0.29 % and 40.97 % ± 0.09 %, respectively, while the control group exhibited the lowest uronic acid content at 12.19 % ± 0.51 %. These findings are consistent with the results of Chen's study (Chen et al., 2019). Therefore, it can be concluded that both microwave pretreatment and extractant-based extraction can increase the uronic acid content of polysaccharides, with those extracted using near-neutral extractants exhibiting higher uronic acid content. A comparison of these results with carbohydrate content shows an inverse relationship between uronic acid and carbohydrate content. This phenomenon has also been observed in studies examining the impact of extraction methods on the chemical properties of SHP (Han et al., 2021). These differences highlight how varying extraction methods lead to changes in the basic composition of SHP.

### 3.3 Monosaccharide composition of SHP

**Fig. 2:**
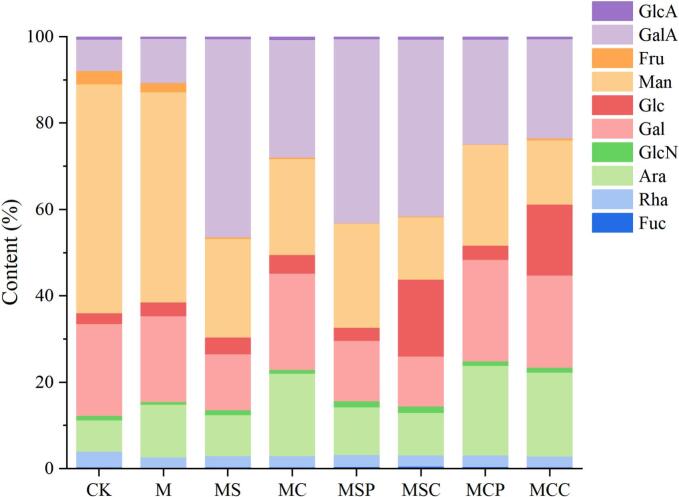
Monosaccharide composition of SHP by different extraction methods. GlcA: glucuronic acid, GalA: galacturonic acid, Fru: fructose, Man: mannose, Glc: glucose, Gal: galactose, GlcN: glucosamine, Ara: arabinose, Rha: rhamnose, Fuc: fucose.

Monosaccharide composition is essential for the structural characterization and biological activity of polysaccharides, with variations in composition resulting in polysaccharides that exhibit unique structures and functions (Bhiri et al., 2024). In this study, the monosaccharide composition of SHP was analyzed, and the results are presented in Fig. 2. The results indicate that the eight polysaccharides are primarily composed of rhamnose (Rha), arabinose (Ara), galactose (Gal), glucose (Glc), mannose (Man), and galacturonic acid (GalA). While the types of monosaccharides in different SHPs display significant homogeneity, their contents vary substantially. In the CK group, Man content is the highest (52.97 %), followed by Gal (21.28 %), Ara (7.29 %), and GalA (7.24 %). Following microwave pretreatment, the mannose content in the M group decreased (48.71 %), while the contents of Ara (12.19 %) and GalA (10.26 %) increased. In the polysaccharides extracted with sodium citrate, specifically MS, MSP, and MSC, the contents of Man (22.82 %, 24.11 %, 14.44 %) and Gal (12.92 %, 13.96 %, 11.59 %) decrease, while the GalA content significantly increases (45.87 %, 42.59 %, 41.00 %). The polysaccharides extracted with citric acid—MC, MCP, and MCC—exhibit a relatively balanced monosaccharide composition, with a notable increase in Ara content (19.15 %, 20.76 %, 19.51 %). Additionally, the study observed changes in the monosaccharide composition when combined with enzyme treatment; the GalA content in MSP and MCP decreases, possibly due to pectinase degrading pectin substances into smaller molecular polymers. MSC and MCC have the lowest Man content among all samples (14.44 %, 14.83 %) and the highest Glc content (17.77 %, 16.42 %). Man is primarily present in hemicellulose-type polysaccharides, and cellulase can hydrolyze β-1,4-D-glucan in cellulose, producing smaller molecules of glucose, cellobiose, and oligosaccharides (Zheng et al., 2024). The enzymes can degrade insoluble substances in cells into soluble carbohydrates, resulting in changes in the composition of polysaccharide monosaccharides (Zhao et al., 2023). Different extraction methods have minimal impact on the monosaccharide composition of polysaccharides but significantly affect their monosaccharide content. Variations in monosaccharide composition may be due to the extraction technique's capacity to induce hydrolytic cleavage of polysaccharide chains and disrupt intermolecular hydrogen bonds (Tang et al., 2023a).

Additionally, the molar ratio of monosaccharides can reveal fundamental structural characteristics of polysaccharide molecules. Based on the monosaccharide composition results, the calculations in this study are shown in Table 2. The linearity indices of MS, MSP, and MSC (1.81, 1.51, 1.67) are higher than those of the other groups, suggesting that polysaccharides extracted with sodium citrate possess more linear structural domains. This may be due to the neutral extractant causing the side chains of neutral polysaccharides to cleave. Ara and Gla are the main components of the side chains in RG-I domains, hence M, MC, MCP, and MCC exhibit higher degrees of branching (12.85, 16.15, 16.41, 16.71). CK, M, and polysaccharides extracted with citric acid are classified as RG-I type pectic polysaccharides, characterized by a high proportion of RG-I (35.86 %, 37.06 %, 46.53 %). In contrast, polysaccharides extracted with sodium citrate are primarily composed of HG structural domains (43.25 %). Therefore, different extraction methods can differentially impact the structure of polysaccharides, with the choice of extractant exerting the most significant influence. In practical applications, the choice of extraction method can be tailored based on the desired monosaccharide composition or content.

**Table 2:** Structural information of SHP by different extraction methods.

Sample	ALinearity	^B^Branching ability	^C^HG (%)	^D^RG-I (%)	^E^DM (%)
CK	0.22	7.84	3.60 %	35.86 %	24.70 %
M	0.30	12.85	7.76 %	37.06 %	29.93 %
MS	1.81	8.54	43.25 %	27.61 %	23.52 %
MC	0.61	16.15	24.61 %	46.53 %	35.08 %
MSP	1.51	8.95	39.79 %	30.60 %	24.45 %
MSC	1.67	8.28	38.41 %	26.69 %	19.68 %
MCP	0.51	16.41	21.60 %	49.62 %	37.13 %
MCC	0.52	16.71	20.50 %	45.80 %	34.05 %

A Linearity: GalA/(Rha + Ara + Gal+Fuc) (Shafie & Gan, 2020); ^B^Branching ability: (Gal+Ara)/Rha; ^C^HG = GalA mol%-Rha mol%; ^D^RG-I = GalA mol%-HG mol% + Rha mol% + Gal mol% + Ara mol%; ^E^DM: Methyl-esterification calculated by FT-IR.

### 3.4 Fourier transform-infrared (FT-IR) analysis

**Fig. 3:**
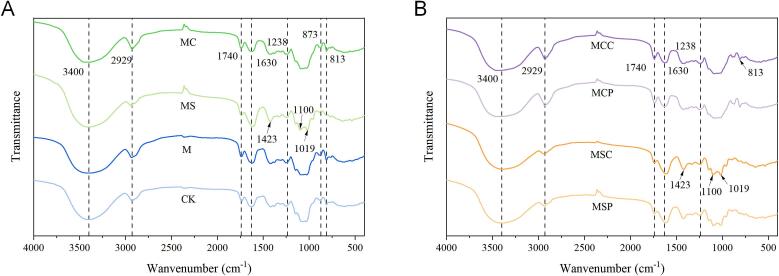
FT-IR spectrum of SHP by different extraction methods. (A) Microwave-assisted extractant extraction; (B) Microwave-assisted extractant-enzyme extraction.

The infrared spectra of polysaccharides extracted by different methods are shown in Fig. 3. From the figure, it can be seen that the characteristic absorption peaks in the infrared spectra of each group are quite similar. The broad peak at 3500–3100 cm^−1^ is attributed to the stretching vibrations of O—H and N—H, suggesting the association of hydroxyl groups within and between polysaccharide molecules. The absorption peak at 3000–2800 cm^−1^ corresponds to the stretching vibration of C—H in carbohydrates. The absorption peaks at 1400–1200 cm^−1^ are from the bending vibration of C—H. These two sets of peaks confirm that the extracts are carbohydrate-based compounds. The range of 2000–1000 cm^−1^ contains specific absorption peaks for pectin substances. The absorption peak at 1740 cm^−1^ is attributed to the ester C=O stretching vibration formed by the carboxyl group of GalA (methyl-esterified galacturonic acid). The peak at 1630 cm^−1^ corresponds to the asymmetric stretching vibration of C=O in carboxyl groups (COO^−^), which is associated with uronic acid residues, indicating the presence of polysaccharide–protein conjugates (Yang et al., 2023a). The DM of the eight polysaccharide fractions ranged from 19.68 % to 37.13 % (Table 2), suggesting that all SHPs can be classified as low-methoxyl pectic polysaccharides (DM < 50 %). The peaks at 1159–1080 cm^−1^ are due to the C—O stretching vibration, indicating the presence of ether bonds within the polysaccharides (Bai et al., 2022). The absorption peak at 873 cm^−1^ is caused by the transverse vibration of methylene groups, and the peak around 813 cm^−1^ is due to the bending vibration of C—H in furan rings, indicating that the majority of the polysaccharides extracted via microwave pretreatment and citric acid are furanose. The infrared spectrum of polysaccharides extracted with sodium citrate displays some differences. The absorption peaks at 1423 cm^−1^ and 1238 cm^−1^ correspond to the C—O stretching vibration of carboxyl groups and the O—H bending vibration, respectively. The absorption peaks between 1010 and 1150 cm^−1^ suggest the presence of pyranose structures, with the peak at 1100 cm^−1^ possibly attributed to the bending vibrations of secondary hydroxyl groups resulting from intramolecular ether C—O stretching. The absorption peak at 1019 cm^−1^ is due to the bending vibration of primary hydroxyl groups in polysaccharides, which are characteristic absorption peaks of pyranose ether bonds and uronic acids. The increased peak intensity at these two points for MS, MSC, and MSC suggests that sodium citrate extraction enhances the content of uronic acids in polysaccharides, consistent with their basic compositional analysis. The absorption peaks at 956 cm^−1^ and 893 cm^−1^ are related to β-type pyranose. Therefore, it can be inferred that polysaccharides extracted with sodium citrate may contain a significant amount of pyranose. In conclusion, microwave pretreatment does not significantly alter the structure or functional groups of polysaccharides, whereas different extraction agents significantly influence polysaccharide structure. Acidic extractants are favorable for extracting furanose, and sodium citrate extraction may enhance the combination of polysaccharides with proteins to form glycoproteins, while also increasing the uronic acid content in polysaccharides. The addition of enzymes minimally affects the structure of SHP.

### 3.5 X-ray diffraction (XRD) analysis

**Fig. 4:**
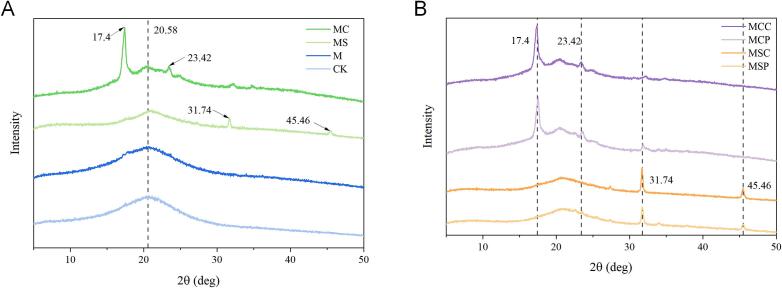
XRD spectrum of SHP by different extraction methods. (A) Microwave-assisted extractant extraction; (B) Microwave-assisted extractant-enzyme extraction.

The X-ray diffraction patterns of SHP extracted by different methods are shown in Fig. 4. The analysis indicates that the structure of SHP comprises both amorphous and crystalline regions. From the figure, it can be observed that all samples exhibit the same broad peak at 2θ 20.58°, which is a characteristic of the amorphous polysaccharide structure. Bai et al. (Bai et al., 2022) also observed similar crystalline peak shapes in chickpea water-soluble polysaccharides and demonstrated that this is a characteristic XRD pattern of semi-crystalline polymers. Samples treated with microwaves exhibit characteristic peaks at 2θ 20.7°, consistent with the CK group. Polysaccharides extracted with sodium citrate (MS) exhibit sharp peaks at 2θ 31.76° and 45.47°, corresponding to characteristic absorption peaks of NaCl. Samples extracted with citric acid show distinct sharp peaks at 2θ 17.4° and 23.42°, which closely align with the characteristic absorption peaks of citric acid and sodium citrate. The results suggest that the crystallinity of polysaccharides significantly increases post-extraction, with different extraction agents causing variations in crystallinity. This increase in crystallinity may result from the introduction of Na^+^ and Cl^−^ ions, which form NaCl and contribute to enhanced crystallinity. In this study, compared to the CK group, the other groups showed a slight decrease in the peak area at 2θ of 20°, along with a slight right shift of the crystalline peaks. These results indicate that microwaves and extracting agents can disrupt the semi-crystalline regions of polysaccharides. The data also reveal that the crystallinity of polysaccharides is mainly influenced by the type of extracting agent, with the type of enzyme having minimal impact. This indirectly confirms that microwave pretreatment combined with extracting agents alters the structure of polysaccharides, consistent with the FT-IR results.

### 3.6 Scanning electron microscopy (SEM) analysis

**Fig. 5:**
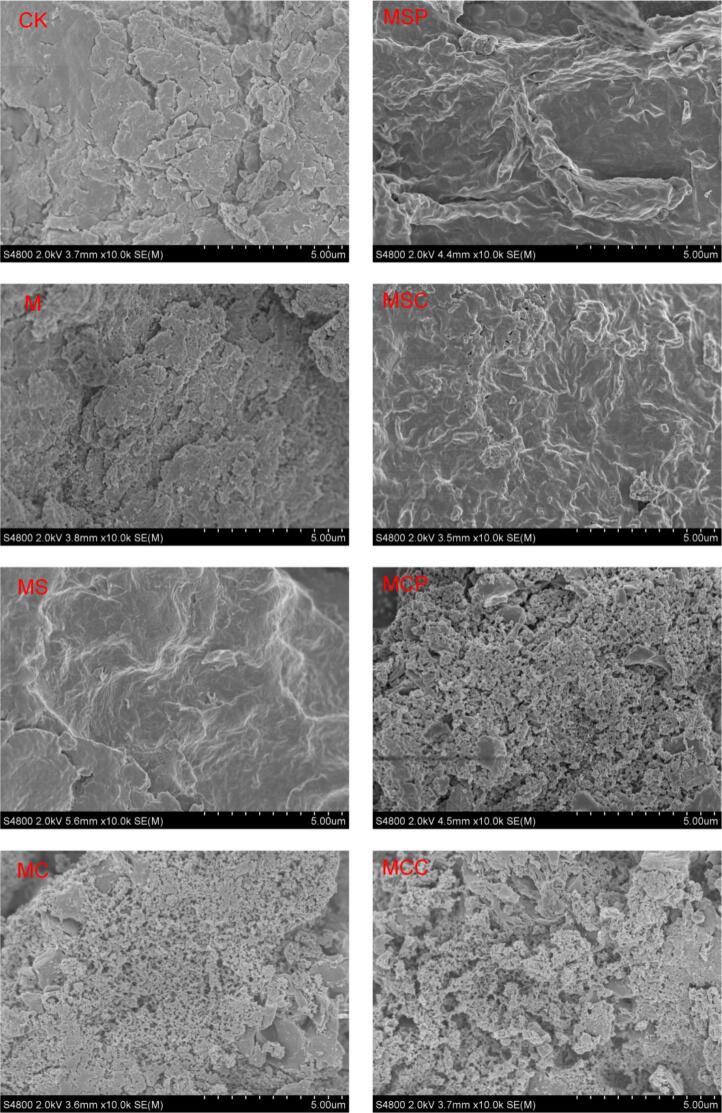
Surface structural characteristics of SHP by different extraction methods.

The structural characteristics of SHP are illustrated in Fig. 5. Significant structural differences were observed among the various treatment groups. The surface morphology of the CK group was smooth and flaky. Following microwave treatment, the sample surface became rough and developed a complex network structure with fragments, potentially due to microwave-induced hydrogen bonding among polysaccharide molecules. Post sodium citrate extraction, the MS surface exhibited a wrinkled and uneven morphology. With the addition of enzymes, the MSC sample displayed more pronounced and relatively flattened folds. These distinct microstructures suggest that the combination of microwave treatment and extraction agents may disrupt the cross-linking between polysaccharide molecules, thereby reorganizing the pectin network structure. In the MC sample, spherical aggregates of varying sizes were observed post-citric acid extraction, indicating that the citric acid extractant modified the polysaccharide structure. These structural differences may result from variations in monosaccharide composition, uronic acid content, and protein content. In the MCP sample, a porous, honeycomb-like gel network structure was observed, suggesting that the addition of pectinase significantly degraded large polysaccharide aggregates. This may be attributed to the strong repulsive forces among polysaccharide molecules obtained through enzyme-assisted extraction, leading to cross-linking and the formation of a network structure (Gao et al., 2024). Tang et al. (Tang et al., 2023a) found that different extraction methods hydrolyzed the sugar chains in dragon fruit stem polysaccharides to varying degrees, resulting in distinct microstructures. The findings demonstrate that microwave pretreatment does not significantly damage the polysaccharide structure, whereas the type of extractant can induce structural alterations in the polysaccharides. The results also suggest that varying extraction methods may influence the degree of sugar chain hydrolysis, thereby altering the apparent morphology of SHP.

### 3.7 Emulsifying properties analysis

The emulsifying activity of polysaccharides primarily originates from the protein fraction, whereas emulsion stability is predominantly attributed to the polysaccharide fraction (Liu, Wu, et al., 2024).

Emulsification of polysaccharides refers to the ability of hydrophilic polysaccharides to form a charged adsorption layer on the surface of oil molecules after mixing with water and oil, thereby creating an emulsion (Yang et al., 2023b). As shown in Table 3, the ranking of emulsion activities of the various SHP in decreasing order was: MS > MCC > MCP > MSP > MC > MSC > CK > M. The emulsifying activity of polysaccharides decreased following microwave pretreatment. This reduction may be attributed to the denaturation of certain proteins and the subsequent decrease in protein content induced by microwave pretreatment, which is consistent with the observed protein content in SHP. MCC and MCP demonstrated excellent emulsifying activity, which was closely related to their molecular structures and DM. These two polysaccharides contained more hydrophobic groups such as methoxy groups and hydrophilic groups such as carboxyl groups, facilitating rapid adsorption at the oil–water interface (Wang et al., 2017). Furthermore, their highly branched architectures enhanced the spreading of molecules across the interface (Qi et al., 2017). The pH of the extraction medium also played a crucial role, as it altered the molar ratio of monosaccharides and the degree of methylation, thereby modulating the interfacial behavior of the polysaccharides. In particular, extraction under a lower pH in citric acid solution may lead to a more compact branched conformation, which in turn contributes to improved emulsifying performance (Bindereif et al., 2022). Lv et al. found that okra polysaccharides extracted with hydrochloric acid at pH 1.0 exhibited higher emulsification capacity when exploring the correlation between physicochemical properties, interfacial behavior, and emulsification characteristics.

**Table 3:** Emulsifying properties of SHP by different extraction methods.

Sample	Emulsifying activity (m^2^/g)	Emulsion Stability (min)
CK	153.43 ± 0.16^e^	682.55 ± 7.34^d^
M	113.97 ± 0.19^g^	752.35 ± 4.24^c^
MS	277.48 ± 0.63^a^	6232.07 ± 14.05^a^
MC	179.34 ± 0.03^d^	4326.30 ± 0.64^b^
MSP	192.75 ± 1.19^c^	283.40 ± 1.81^f^
MSC	136.48 ± 1.99^f^	112.83 ± 3.85^h^
MCP	197.94 ± 1.65^b^	317.95 ± 7.48^e^
MCC	199.70 ± 1.01^b^	230.24 ± 10.03^g^

Data represent mean ± standard deviation (n = 3), and different letters indicate significant differences (p < 0.05) between different extractions.

Compared with other samples, MS exhibited the highest emulsifying activity and stability, which was directly related to the introduction of Na^+^. The emulsifying activity of polysaccharides primarily depends on the protein moieties, whereas the stability of the emulsion is mainly attributed to the polysaccharide fraction (Liu, Wu, et al., 2024). Low-methoxyl pectic polysaccharides possess a large number of free carboxyl groups (COO^−^), whose strong electrostatic repulsion causes the polysaccharide chains to extend, hindering effective adsorption at the oil–water interface (Fan, 2023). Na^+^ can interact with the charged groups, such as carboxyl groups, partially shielding the electrostatic repulsion and promoting the oriented alignment and tight adsorption of polysaccharide molecules at the interface (Yang, Wang et al., 2024). In addition, the salting-in effect can modulate the surface charge distribution of proteins and the solvation of zwitterionic groups, thereby enhancing protein solubility and further improving the emulsifying properties of the polysaccharides (Sarkar et al., 2016). Moreover, an appropriate concentration of Na^+^ can enhance the linearity and solubility of polysaccharide molecules, facilitating the formation of a more uniform interfacial film and thus improving emulsion stability (Zhang et al., 2023).

### 3.8 Viscosity analysis

**Fig. 6:**
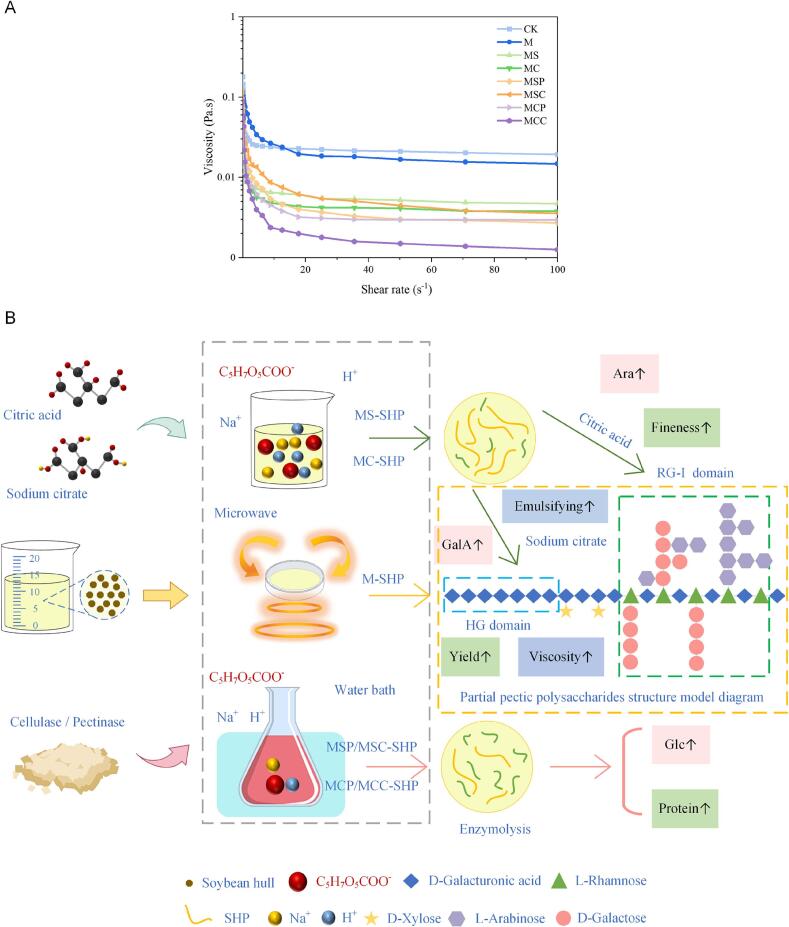
Effect of different extraction methods on the functional properties of SHP. (A) Apparent viscosity of SHP by different extraction methods; (B) Schematic representation of different methods of polysaccharide extraction from soybean hulls.

Polysaccharides are widely used in food processing, which is likely related to their gel-forming properties. Studies have shown that the gelling characteristics of polysaccharides depend on apparent viscosity (Liu, Wang et al., 2024); therefore, the apparent viscosity of polysaccharide samples extracted using different methods was measured. The apparent viscosity of various polysaccharide samples was measured. As illustrated in Fig. 6, the apparent viscosity of all samples decreases with increasing shear rate, demonstrating shear-thinning behavior. The measured data were fitted using the Power Law model y = Kx^n^, where “K” is the consistency coefficient representing the viscosity of the liquid, and “n” is the flow behavior index, indicating the deviation of the sample fluid from Newtonian behavior (Yang, Zhang, et al., 2020), with the results presented in Table 4. The flow index (n) for all samples is less than 1, indicating that the polysaccharide samples exhibit pseudoplastic fluid behavior. According to the consistency coefficient (K), the apparent viscosity of the samples follows the order: M480 > CK > MSC > MS > MSP > MC > MCP > MCC. The apparent viscosity of polysaccharides is related to the total sugar content of SHP. This result indirectly indicates that microwave pretreatment can enhance the total sugar content of SHP, which is consistent with the results shown in Fig. 2. Furthermore, microwave pretreatment may enhance interactions between polysaccharide molecules, inhibiting SHP flow behavior and thereby increasing sample viscosity. The rheological properties of pectic polysaccharides are also influenced by the distribution of substituents and the degree of branching. The apparent viscosity of polysaccharides extracted with sodium citrate was generally higher than that of those extracted with citric acid, possibly because Na^+^ ions replaced certain groups within the polysaccharide, inducing SHP gelation and thereby increasing apparent viscosity. Based on the results in Table 2, it can be concluded that microwave pretreatment and citric acid extraction lead to a higher degree of branching in SHP, which enhances the viscosity of the sample. However, the low pH during citric acid extraction may cause the breaking of hydrogen bonds between polysaccharide molecules, resulting in structural changes and a decrease in the viscosity of the polysaccharide solution (Wang et al., 2019). These findings confirm that SHP has the potential for application in gel formation.

**Table 4:** Parameters of power law equation for polysaccharide extraction by different methods.

Sample	K (Pa·s^n^)	n	R^2^
CK	0.0309 ± 0.0008^b^	0.8995 ± 0.0061^a^	0.9997
M	0.0423 ± 0.0028^a^	0.7675 ± 0.0158^c^	0.9968
MS	0.0086 ± 0.0003^d^	0.8688 ± 0.0095^ab^	0.9992
MC	0.0063 ± 0.0004^de^	0.8856 ± 0.0159^a^	0.9977
MSP	0.0086 ± 0.0006^d^	0.7476 ± 0.0172^c^	0.9971
MSC	0.0234 ± 0.0012^c^	0.5794 ± 0.0122^e^	0.9965
MCP	0.0058 ± 0.0005^e^	0.8472 ± 0.0206^b^	0.9956
MCC	0.0050 ± 0.0006^e^	0.6967 ± 0.0301^d^	0.9932

“K” is the coefficient of consistency, which is equivalent to a viscosity measurement, “n” is the flow index, and the R-value indicates the degree to which the fluid deviates from a Newtonian fluid. Data represent mean ± standard deviation (n = 3), and different letters indicate significant differences (p < 0.05) between different extractions.

## 4 Conclusion

The purpose of this study was to investigate the impact of microwave pretreatment combined with extractant and enzymatic extraction on the structural and functional properties of SHP. In comparison with CK, microwave pretreatment effectively increased the extraction yield and carbohydrate content of SHP, while moderately reducing its protein content. Although the monosaccharide composition of different SHPs was relatively consistent, their relative abundances varied considerably. SHP primarily consisted of rhamnose, arabinose, galactose, and mannose. SHP extracted using microwave pretreatment combined with sodium citrate (MC, MCP and MCC) exhibited greater linearity, whereas SHP extracted with citric acid (MS, MSP and MSC) showed a higher degree of branching. Additionally, analysis of SHP's functional properties indicated that MS-extracted polysaccharide exhibited superior emulsifying activity and emulsion stability compared to others. The emulsifying properties of polysaccharides are strongly governed by their structural features, including component composition (etc. protein and polysaccharide), branching architecture, degree of methyl esterification, and sodium ion presence. These findings provide theoretical support for the efficient extraction of SHP and its potential applications in the food and pharmaceutical industries, laying the foundation for the future development of plant polysaccharides with enhanced structural and functional properties.

## CRediT authorship contribution statement

**Rui Zhang:** Writing – review & editing, Writing – original draft, Validation, Software, Methodology, Data curation, Conceptualization. **Daozi Deng:** Validation, Software. **Hong Song:** Writing – review & editing. **He Liu:** Writing – review & editing, Resources, Funding acquisition. **Shuai Yang:** Resources.

## Declaration of competing interest

The authors declare that they have no known competing financial interests or personal relationships that could have appeared to influence the work reported in this paper.

## Data Availability

Data will be made available on request.
